# Stimulatory effect of *Eucalyptus *essential oil on innate cell-mediated immune response

**DOI:** 10.1186/1471-2172-9-17

**Published:** 2008-04-18

**Authors:** Annalucia Serafino, Paola Sinibaldi Vallebona, Federica Andreola, Manuela Zonfrillo, Luana Mercuri, Memmo Federici, Guido Rasi, Enrico Garaci, Pasquale Pierimarchi

**Affiliations:** 1 Institute of Neurobiology and Molecular Medicine – ARTOV, CNR, Via Fosso del Cavaliere 100, 00133 Rome, Italy; 2 Department of Experimental Medicine and Biochemical Science, University of Rome "Tor Vergata", Via Montpellier 1, 00133, Rome, Italy; 3 INAF-IASF, Via Fosso del Cavaliere 100, 00133 Rome, Italy

## Abstract

**Background:**

Besides few data concerning the antiseptic properties against a range of microbial agents and the anti-inflammatory potential both *in vitro *and *in vivo*, little is known about the influence of *Eucalyptus *oil (*E*O) extract on the monocytic/macrophagic system, one of the primary cellular effectors of the immune response against pathogen attacks. The activities of this natural extract have mainly been recognized through clinical experience, but there have been relatively little scientific studies on its biological actions. Here we investigated whether *E*O extract is able to affect the phagocytic ability of human monocyte derived macrophages (MDMs) *in vitro *and of rat peripheral blood monocytes/granulocytes *in vivo *in absence or in presence of immuno-suppression induced by the chemotherapeutic agent 5-fluorouracil (5-FU).

**Methods:**

Morphological activation of human MDMs was analysed by scanning electron microscopy. Phagocytic activity was tested: i) *in vitro *in *EO *treated and untreated MDMs, by confocal microscopy after fluorescent beads administration; ii) *in vivo *in monocytes/granulocytes from peripheral blood of immuno-competent or 5-FU immuno-suppressed rats, after *EO *oral administration, by flow cytometry using fluorescein-labelled *E. coli*. Cytokine release by MDMs was determined using the BD Cytometric Bead Array human Th1/Th2 cytokine kit.

**Results:**

*E*O is able to induce activation of MDMs, dramatically stimulating their phagocytic response. *E*O-stimulated internalization is coupled to low release of pro-inflammatory cytokines and requires integrity of the microtubule network, suggesting that *E*O may act by means of complement receptor-mediated phagocytosis. Implementation of innate cell-mediated immune response was also observed *in vivo *after *E*O administration, mainly involving the peripheral blood monocytes/granulocytes. The 5-FU/*EO *combined treatment inhibited the 5-FU induced myelotoxicity and raised the phagocytic activity of the granulocytic/monocytic system, significantly decreased by the chemotherapic.

**Conclusion:**

Our data, demonstrating that *Eucalyptus *oil extract is able to implement the innate cell-mediated immune response, provide scientific support for an additional use of this plant extract, besides those concerning its antiseptic and anti-inflammatory properties and stimulate further investigations also using single components of this essential oil. This might drive development of a possible new family of immuno-regulatory agents, useful as adjuvant in immuno-suppressive pathologies, in infectious disease and after tumour chemotherapy.

## Background

The monocytic/granulocytic system as well as differentiated macrophages constitute the primary cellular effectors of the immune response, playing a pivotal role in the detection and elimination of foreign bodies such as pathogenic microorganisms. Recognition of foreign microorganisms by these cells ultimately results in phagocytosis and in the eventual destruction of pathogens by lysosomal enzymes. Macrophages perform a variety of functions other than phagocytosis [1]. The phagocytic process is accompanied by intracellular signals that trigger cellular responses such as cytoskeletal rearrangement, alterations in membrane trafficking, activation of apoptosis, release of chemical mediators such as growth factors, pro- and anti-inflammatory cytokines and chemokines [2]. Such mediators, produced by activated macrophages, are essential for microbe killing but also potentiate inflammatory reactions; thus regulation of this production is therefore critical to kill pathogens without inducing tissue injury [3].

Natural oils are extensively used in cosmetics as well as in folk medicine for the treatment of a growing number of more or less specific pathologies. Recently the clinical use of essential oils has expanded worldwide also including therapy against various kinds of inflammatory diseases, such as allergy, rheumatism and arthritis. These activities have mainly been recognized through clinical experience, but there have been relatively little scientific studies on biological actions of these natural extracts. For instance, the tea tree oil as well as *Eucalyptus *oil have been demonstrated to possess antiseptic properties against a range of bacteria [4-8] and have been used in a number of products against oral pathogens and different forms of infections. It has also been suggested that tea tree [9,10] and lavender oils [11] are able to suppress allergic symptoms through the inhibition of histamine release [12,13] and cytokine production [14] in *in vitro *and *in vivo*. Monoterpenoid components of aromatic constituents of *Eucalyptus *oil are traditionally used as analgesic, anti-inflammatory, and antipyretic remedies and are commercially available for the treatment of the common cold and other symptoms of respiratory infections. Phytochemical analysis has shown that the profile of the monoterpenoids changes among the *Eucalyptus *species, with potential variations in therapeutic properties. In *Eucalyptus globulus*, the major monoterpenoid component is eucalyptol (1,8-cineole), constituting the 60–90% [15], that has been reported to inhibit the production and synthesis of tumour necrosis factor-α (TNF-α), interleukin-1β (IL-1β), leukotriene B4, and thromboxane B2 in human blood monocytes [15,16]. An anti-inflammatory activity of eucalyptol in patients with bronchial asthma has been also described in a double-blind, placebo-controlled trial [17]. These findings provide scientific support at least for some of the traditionally accepted uses, in folk medicine, of essential oils, and in particular of *Eucalyptus *oil, although a direct relationship is still to be demonstrated.

To our knowledge, actually there are no available data concerning the influence of *Eucalyptus *essential oil on the cellular components of the immune system, the only exception is for the effect on some cytokine production [15,16]. The aim of the present study has been to investigate whether essential oil from *Eucalyptus globulus *(*E*O) is able to affect the phagocytic activity of human monocyte-derived macrophages (MDMs) *in vitro *and of peripheral blood monocytes/granulocytes from immuno-competent rats treated *in vivo *in absence or in presence of immuno-suppression induced by administration of the chemotherapeutic agent 5-fluorouracil (5-FU).

## Results

### *In vitro *effect of *Eucalyptus *oil on morphological features and phagocytic activity of human MDMs

**Figure 1:**
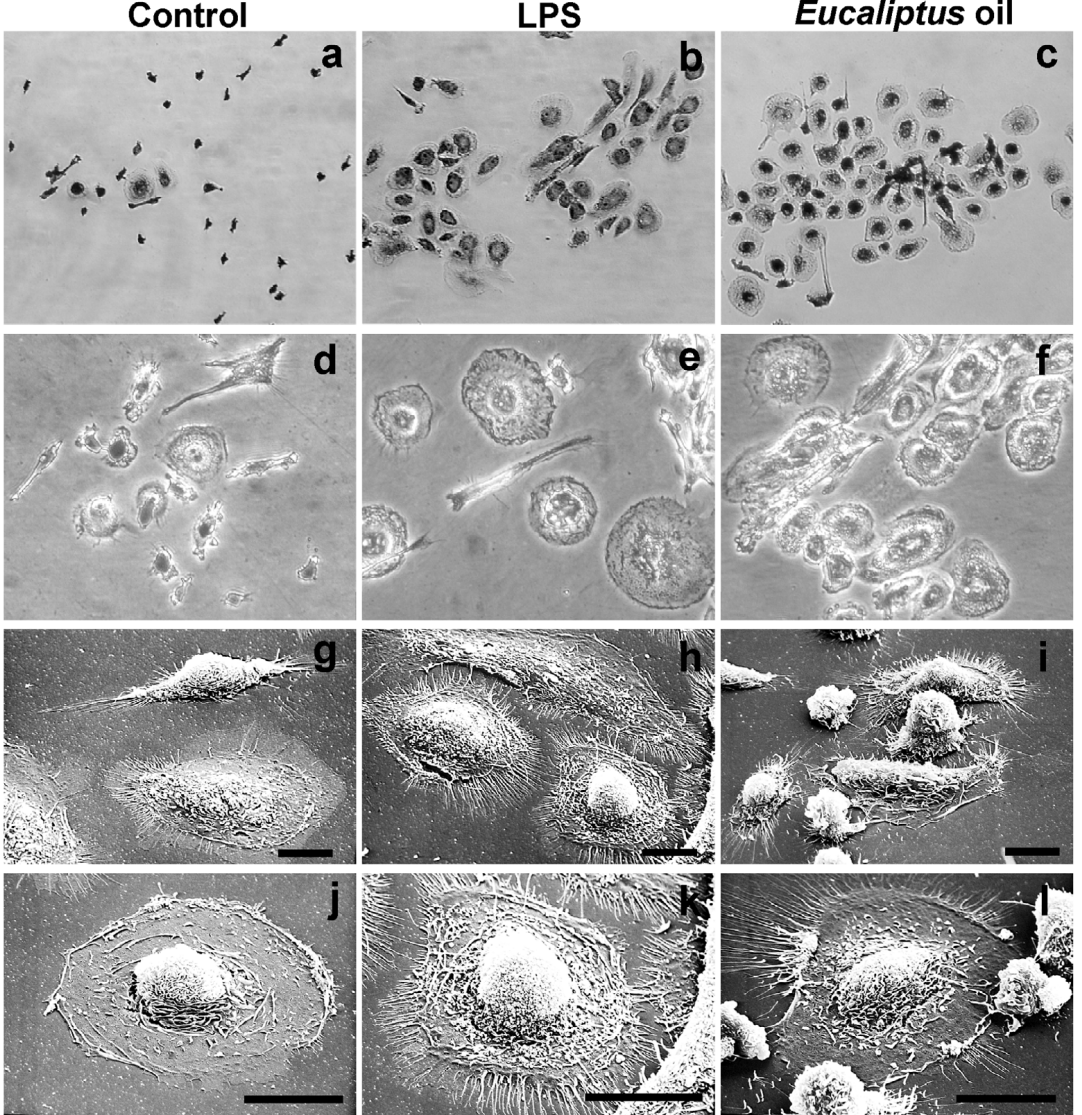
**Morphological features of human MDMs after 24 h of *in vitro *treatment with *Eucalyptus *oil**. **a**-**f**, phase-contrast microscopy after Wright Giemsa staining: **a**, **d**, untreated control; **b**, **e**, MDMs stimulated with 0.1 μg/ml of bacterial lipopolysaccharide (LPS); **c**, **f**, MDMs treated with 0.016% *Eucalyptus *oil; **a**, **b**, **c**, original magnification: 20×;**d**, **e**, **f**, original magnification: 40×. **g**-**l**, Scanning electron microscopy of untreated (**g**, **j**), LPS treated (**h**, **k**) and *Eucalyptus *oil treated MDMs (**i**, **l**). *Bars*: 20 μm.

**Figure 2:**
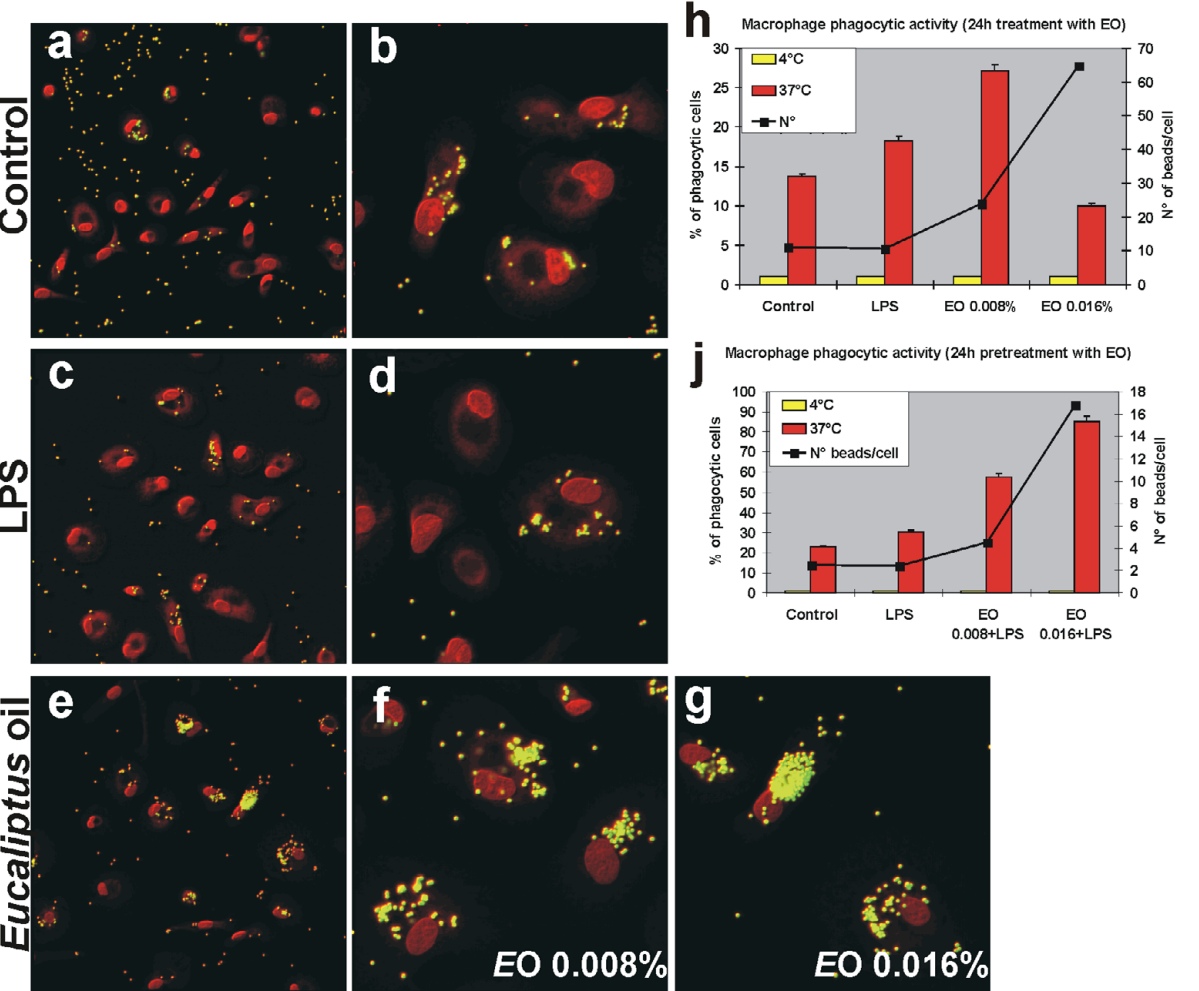
***In vitro *effect of *Eucalyptus *oil treatment on phagocytic activity of human MDMs**. The phagocytic activity of treated and untreated MDMs was tested by adding to cultures 2 × 10^7 ^beads/ml of fluorescent polystyrene beads as described in Methods. **a**-**g**, confocal microscopy images, showing the beads uptake (yellow-green hue) in control (**a**, **b**), LPS treated (**c**, **d**) and *E*Otreated MDMs (**e**-**g**); cells were counter-stained with 1 μg/ml propidium iodide (red hue). **a**, **c**, **e**, Original magnification: 40×; **b**, **d**, **f**, **g**, original magnification: 100×. **h**, **j**, Evaluation of phagocytic activity performed counting the number of phagocytic MDMs, reported as percent of phagocytic cells (solid bars), as well as the number of beads per cell (■). A minimum of 500 cells per sample were observed. Evaluation of percentage of phagocytic cells in controls maintained at 0°C, to block beads internalization, is also reported (yellow solid bars).

*E*O treatment, at both concentrations used in our experiments, did not affect the macrophage viability, as revealed by the Trypan blue exclusion method (see Additional file 1). Morphological observation of human MDMs (Fig. 1) showed that after 24 h treatment with 0.008% or 0.016% *EO*, cells assumed the typical activated morphology, also exhibited by LPS-activated MDMs, showing an enlarged size, more abundant microvillous structures as compared to the untreated macrophages, a rougher surface with prominent filopodia, blebs, and rufflings. Confocal microscopic observation, after fluorescent beads administration to cell cultures (Fig. 2), showed that *E*O is able to dramatically increase, in a dose dependent manner, the phagocytic activity of MDMs, to higher extent compared to the LPS treatment. In fact, as reported in Fig. 2h, in untreated control cultures 13.7% of cells were phagocytic, with a mean of 11 beads phagocytosed per cell. In cultures treated for 6 h with LPS, the percentage of phagocytic cells is lightly increased (18.26%), while no increase were recorded in the mean number of beads/cell. Conversely, in sample 24 h treated with 0.008% *E*O, the percentage of phagocytic cells increases to 27.1% and the average number of phagocytosed beads/cell raises to 24. The treatment with the highest concentration of *E*O (0.016%), increased so dramatically the phagocytic activity of MDMs, showing up to 64.8 phagocytosed beads/cell, that the majority of cells engulfed with indigestible beads possibly died, leading to a registration of only 10% of phagocytic cells. Moreover, pre-treatment with *E*O 24 h before the addition of LPS to cell culture was able to increase, without any cytotoxicity and in a dose-dependent manner, the phagocytic activity of human MDMs in comparison with the treatment with LPS alone (Fig. 2j).

We excluded a non specific effect on macrophage phagocytic activity caused by the oil preparation, by testing other two oil extracts, *Lavender *oil (*Lav*O) and the *Tea Tree *oil (*TeaTree*O) in parallel with *E*O. The results obtained (see the Additional file 2), showed that treatment with both *Lavender *and *Tea Tree *oil extracts do not affect MDMs phagocytic activity, also confirming the dose-dependent stimulatory effect of *Eucalyptus *oil. Cell viability (see the Additional file 3) showed that *Lav*O, similarly to *E*O, resulted not toxic, indicating that the lack of the effect on MDMs phagocytic ability observed is not ascribable to oil cytotoxicity. Instead, a moderate effect on cell viability of the *TeaTree*O extract has been recorded.

**Figure 3:**
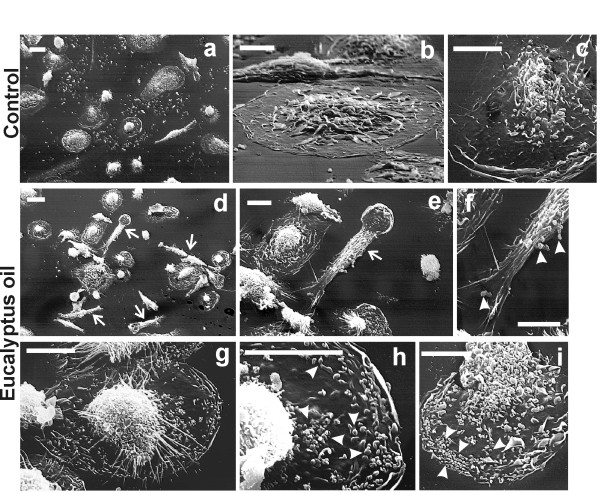
**Morphological features of 24 h *E*O treated human MDMs after *in vitro *administration of polystyrene beads**. Scanning electron microscopy of untreated (**a**-**c**) and 0.008% *Eucalyptus *oil treated MDMs (**d**-**i**) showing the presence, in *E*O treated cultures, of numerous polarised cells exhibiting elongated lamellopodia and filopodia (arrows in panels **d **and **e**), indicative of a pseudopodial activity. Arrowheads in panels **f**, **h**, **i **point to phagocytosed beads, more numerous respect to the untreated control (**c**). *Bars*: **a**, **d **= 20 μm; remaining panels = 10 μm.

Scanning electron microscopy observation showed, after beads addition to *E*O treated cultures, the presence of numerous polarised cells exhibiting elongated lamellopodia and filopodia (arrows in Fig. 3d, e), indicative of a pseudopodial activity. In cell cytoplasm of the *E*O activated MDMs, a higher number of phagocytosed beads (arrowheads in Fig. 3f, h, i), compared to the untreated control (Fig. 3c) were observed, also distributed at the filopodial and lamellopodial structures, suggesting that an active cell motility might contribute to the increased phagocytic ability towards the foreign bodies.

### Effect of *Eucalyptus *oil on production of pro-inflammatory and immune-modulating cytokines by human MDMs

**Figure 4:**
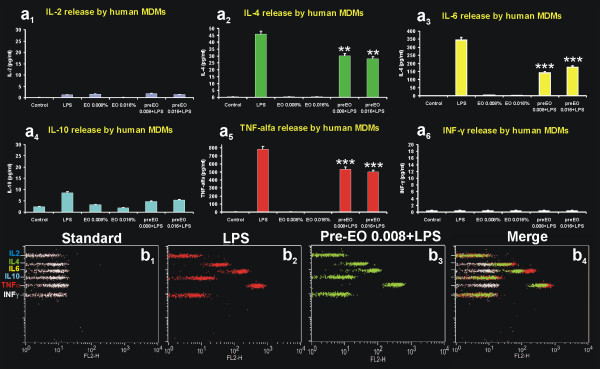
**Th1/Th2 cytokines produced by human MDMs after 24 h of *in vitro *treatment with *Eucalyptus *oil**. **a**, Analysis by cytometric bead array of IL-2 (**a**_1_), IL-4 (**a**_2_), IL-6 (**a**_3_), IL-10 (**a**_4_), TNF-α (**a**_5_), and IFN-γ (**a**_6_) production in untreated controls, LPS stimulated MDMs and cells treated with 0.008% and 0.016% *E*O or pre-treated with *E*O before LPS stimulation. **b**, Cytofluorimetric dot plots showing the modification of cytokines profile in culture medium of MDMs LPS-stimulated (**b**_2_) or pre-treated with *E*O before LPS stimulation (**b**_3_); **b**_4_, merged dot plot showing the decrement in IL-4, IL-6 and TNF-α concentration induced by *E*O pre-treatment; **b**_1_, cytokines profile in standard. **P <0.001 *vs *LPS stimulation; ***P < 0.0001 *vs *LPS stimulation.

In spite of the morphological features typical of activated macrophages, and of the increased phagocytic ability acquired by *E*O-treated or pre-treated MDMs, the release in the extracellular medium of pro-inflammatory and immune-modulating cytokines was significantly lower compared to that recorded for LPS treatment alone (Fig. 4). This effect is particularly evident for the pro-inflammatory cytokines conspicuously produced by MDMs under LPS stimulation, such as IL-4, IL-6 and TNF-α. In detail, in absence of LPS stimulation, *E*O activated macrophages produced very low levels, comparable to the untreated control, of IL-4, IL-6 and TNF-α (Fig. 4a_2_, a_3_, a_5_). Moreover, in accordance with the anti-inflammatory properties ascribed to *E*O, pre-treatment with *E*O 24h before the addition of LPS to cell culture was able to significantly decrease the LPS-induced cytokines production (Fig. 4a_2_, a_3_, a_5_, b). No significant effect on the production of the other immune-modulating cytokines tested (IL-2, IL10 and IFNγ) was recorded for both *E*O or LPS treatments (Fig. 4a_1_, a_4_, a_6_).

### Cytoskeletal elements mediating the *Eucalyptus *oil stimulated internalization

**Figure 5:**
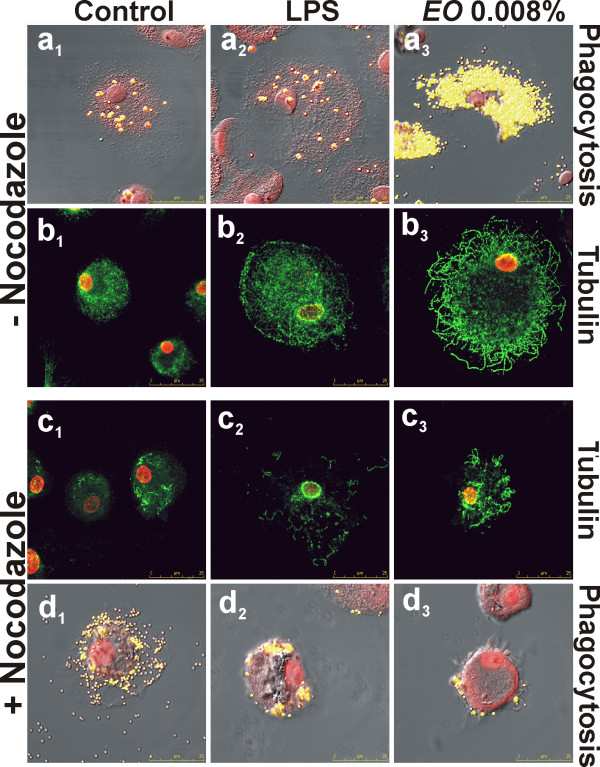
**Effect of microtubule destabilization by nocodazole on phagocytic activity of *Eucalyptus *oil stimulated human MDMs**. **a**, **d**, Confocal microscopy images, showing the beads uptake (yellow hue) in control (**a**_1_, **d**_1_), LPS pre-treated (**a**_2_, **d**_2_) and *E*O pre-treated MDMs (**a**_3_, **d**_3_), in absence (**a**) or in presence (**d**) of nocodazole treatment; cells were counter-stained with PI (red hue). Merged images with differential interference contrast, used to visualize cell morphology, are shown. **b**, **c**, Confocal microscopy images, showing the microtubular network (green hue) in nocodazole treated and untreated cells: **b**_1_, **c**_1_, controls; **b**_2_, **c**_2_, LPS pre-treated MDMs; **b**_3_, **c**_3_, *E*O pre-treated MDMs.

We also investigated whether the observed differences in LPS- or *E*O-induced internalization and cytokine production were associated with differences in the cytoskeletal elements that mediate phagocytosis. In particular, we verified if the phagocytic ability of LPS- or *E*O- stimulated MDMs required integrity of the microtubular network. To this purpose, control cells and macrophages, pre-treated with LPS or *E*O, were treated with the microtubule-destabilizing agent nocodazole and the phagocytic ability was tested after fluorescent beads administration. Confocal microscopic observation (Fig. 5) showed that, after damage of the microtubular network by nocodazole treatment (Fig. 5c), while controls and LPS-stimulated MDMs were still able to internalize beads (Fig. 5d_1_, d_2_), in *E*O-stimulated cells bead uptake was almost completely inhibited (Fig. 5d_3_). These results clearly suggest that LPS- and *E*O-induced phagocytosis occur possibly by means of different mechanisms, since only the *E*O-stimulated internalization requires integrity of the microtubule network.

Moreover, as shown in Fig. 5b, *E*O stimulation is able to provoke a more dramatic reorganization of microtubular filaments respect to LPS, strongly suggesting that tubulin plays an important role in the *E*O-induced macrophage activation.

### *In vivo *effect of *Eucalyptus oil *on activation of peripheral blood mononuclear cells from immuno-competent rats

**Figure 6:**
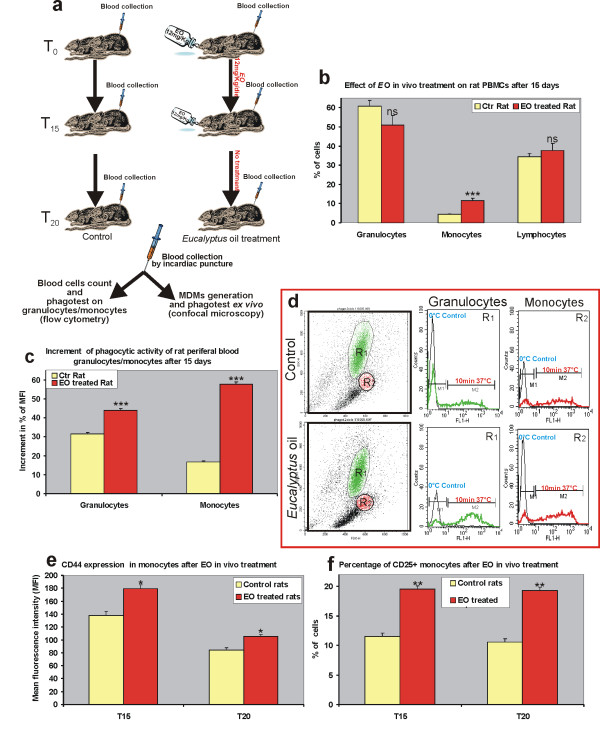
***In vivo *effect of *Eucalyptus *oil on activity of peripheral blood mononuclear cells from immuno-competent rats**. **a**, Schematic diagram of rats treatment, detailed in Methods. **b**, Evaluation by flow cytometry of the percentage of circulating granulocytes, monocytes and lymphocytes in untreated control (yellow solid bar) and in rats after 15 days of *E*O administration (red solid bars). **c**, **d**, Cytofluorimetric evaluation, by phagotest kit, of phagocytic activity of granulocytes and monocytes from untreated control (yellow solid bar) and from rats after 15 days of *E*O administration (red solid bars). The number of internalized bacteria has been recorded as Mean Fluorescence Intensity (MFI): mean values of 12 animals/group are reported as percent of increment of MFI at T_15 _respect to T_0_. Representative plots (**d**) of untreated controls and *Eucaliptus *oil treated animals are also shown; R_1_: granulocyte population, R_2_: monocyte population; phagocytic activity in controls maintained at 0°C, to block *E. coli *bacteria internalization, is also reported (grey lines). **e**, Cytofluorimetric analysis of expression of CD44, reported as MFI, in treated and untreated rats at the end (T_15_) and after 5 days from the end of *E*O administration (T_20_). **f**, Cytofluorimetric evaluation of percentage of CD25^+ ^monocytes in treated and untreated rats at T_15 _and at T_20_. *P < 0.01 *vs *control; **P < 0.001 vs control; ***P < 0.0001 *vs *control; ns = *P *not significative.

We firstly assessed the effect of *E*O on peripheral blood mononuclear cells (PBMCs) after *in vivo *administration to immuno-competent animals, following the scheme of treatment reported in Fig. 6a. The evaluation of haematological parameters at T_15 _revealed that *E*O treatment was able to significantly increase the percentage of circulating monocytes in peripheral blood of treated rats compared to the untreated controls, while no significant modification in the percentage of circulating granulocytes and lymphocytes was evidenced (Fig. 6b). Concomitantly, an increment in the phagocytic activity of granulocytes, and at higher extent of monocytes, obtained from treated animals, was recorded (Fig. 6c, d).

We also examined the monocytic/granulocytic fraction for the expression of surface molecules that may be indicative of leucocytes activation. In particular we evaluated the expression of CD44, the receptor for the extracellular matrix component hyaluronan that mediates the leukocyte/endothelial interactions leading to extravasation [18], and of CD25, a marker of circulating monocyte activation [19,20]. Cytofluorimetric analysis showed that at T_15 _*E*O treatment induced a significant increase of the expression of CD44, as well as the percentage of CD25^+ ^cells in circulating monocytes compared to the untreated control rats, and this effect also persisted at T_20_, after 5 days from the end of *E*O administration (Fig. 6e, f). No significant modification in the expression of CD44 or in the percentage of CD25^+ ^cells was evidenced in circulating granulocytes and lymphocytes (not shown).

**Figure 7:**
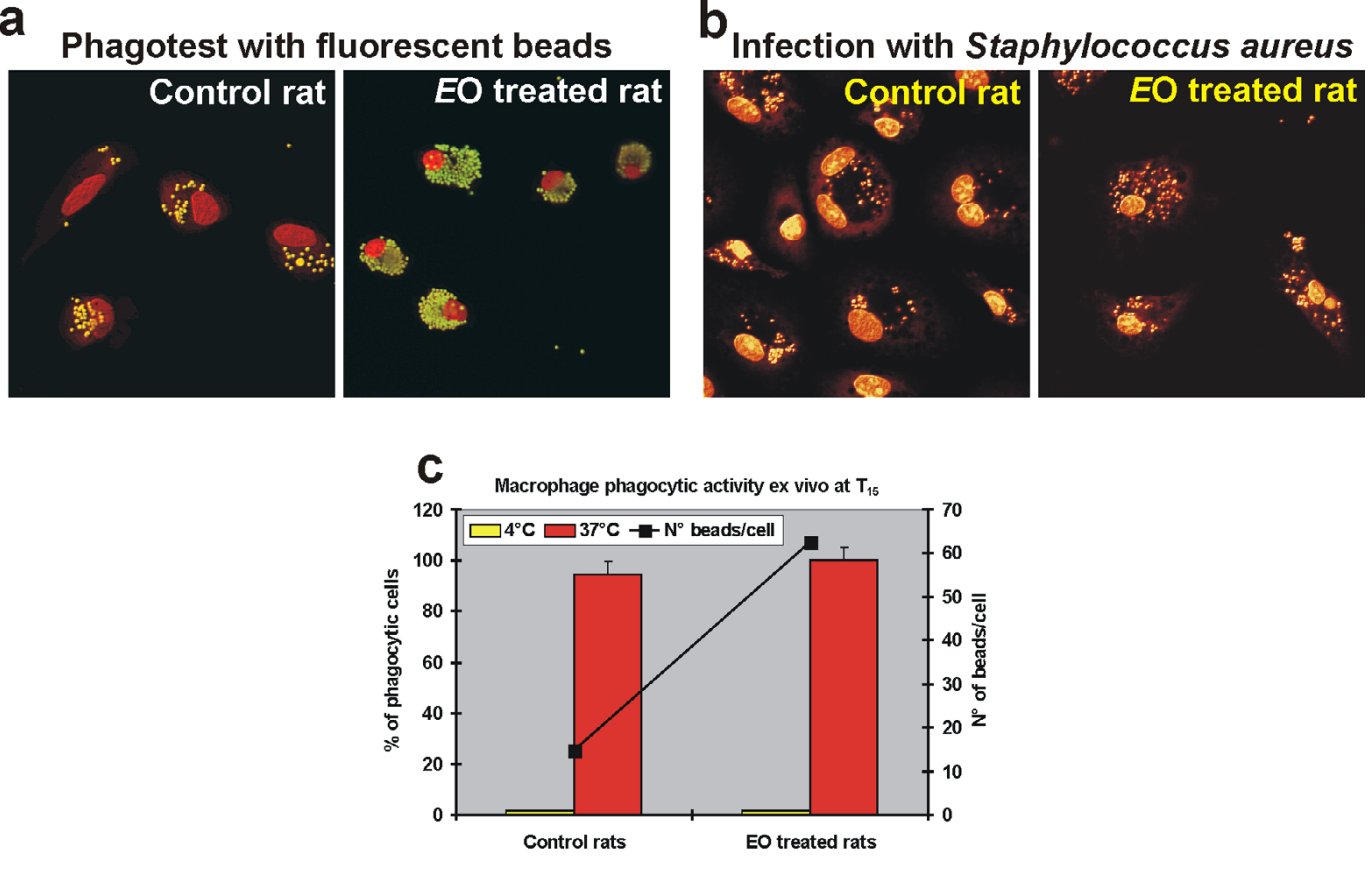
***Ex vivo *evaluation of the phagocytic activity of MDMs obtained from *E*O treated and untreated rats**. Confocal microscopy images showing the uptake of fluorescent beads (**a**) or of *S. aureus *bacteria (**b**) by MDMs obtained from *E*O treated and untreated rats at T_15_; (**b**) *S. aureus *bacteria were visible in cell cytoplasm after PI staining; original magnification: 100×. (**c**) Evaluation of phagocytic activity of rat MDMs performed *ex vivo *counting the number of phagocytic MDMs, reported as percent of phagocytic cells (solid bars), as well as the number of beads per cells (■). The percentage of phagocytic cells in controls maintained at 0°C, to block beads internalization, is also reported (yellow solid bars).

*Ex vivo *evaluation of the phagocytic activity of monocytes derived macrophages (MDMs) obtained from treated and untreated animals at T_15_, revealed that the stimulatory effect recorded in circulating monocytes was also retained in differentiated macrophages (Fig. 7a, c). The *ex vivo *phagocytic ability of MDMs obtained from *E*O treated rats was increased not only towards a non-specific foreign bodies such as fluorescent beads, but also towards microbiological pathogens, as observed after infection with *Staphylococcus aureus *(Fig. 7b).

### *In vivo *effect of *Eucalyptus *oil on phagocytic activity of peripheral blood mononuclear cells from immuno-suppressed rats

**Figure 8:**
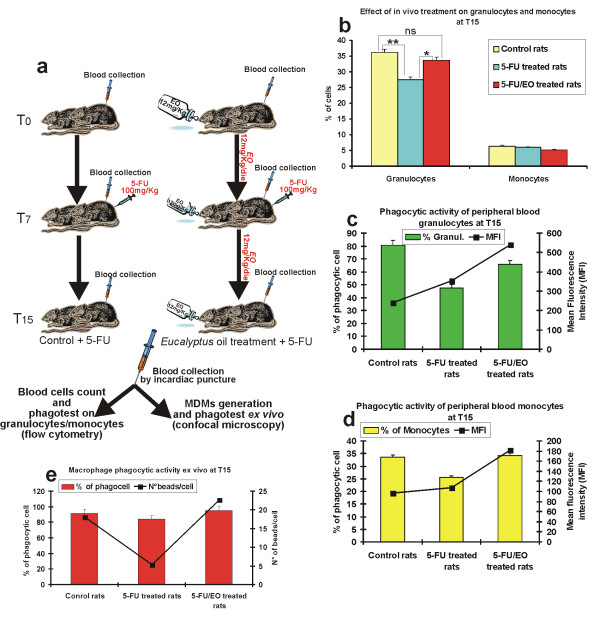
***In vivo *effect of *E*O on activity of peripheral blood mononuclear cells from 5-FU immuno-suppressed rats**. **a**, Schematic diagram of rats treatment, detailed in Methods. **b**, Evaluation at T_15 _of the percentage of circulating granulocytes and monocytes in untreated control (yellow solid bar), in 5-FU immuno-suppressed rats (cyan solid bar) and in rats after 5-FU/*E*O combined administration (red solid bars). **c**, **d**, Cytofluorimetric evaluation of phagocytic activity of granulocytes (**c**) and monocytes (**d**) from untreated control, 5-FU immuno-suppressed rats and rats after 5-FU/*E*O combined administration; the percentage of phagocytic cells (solid bars), as well as the MFI (■), indicative of the number of internalized bacteria/cells, are reported. **e**, Evaluation of phagocytic activity of rat MDMs performed *ex vivo *counting the number of phagocytic MDMs, reported as percent of phagocytic cells (solid bars), as well as the number of beads per cells (■).*P < 0.01; **P < 0.001; ns = *P *not significative.

Finally we verified whether *E*O treatment could be also able to induce a recovery of peripheral blood mononuclear cells activity after bone marrow suppression induced by 5-fluorouracil (5-FU), a chemotherapeutic agent extensively used in the treatment of different types of cancer [21,22] producing myelotoxicity as side effect [23]. The schematic diagram of treatment is reported in Fig. 8a. The evaluation of haematological parameters at T_15 _revealed that *E*O treatment was able to induce a recovery of the percentage of circulating granulocytes that was significantly reduced by 5-FU treatment, while no modification was recorded in the percentage of circulating monocytes, that, on the other hand, was not influenced by the chemotherapeutic agent (Fig. 8b).

5-FU treatment also induced a decrease of phagocytic activity of peripheral blood granulocytes/monocytes, but in *E*O/5-FU treated animals the granulocytes/monocytes phagocytic ability was restored to values near to that of untreated immuno-competent rats (Fig. 8c, d). Finally, the *ex vivo *experiments revealed that the recovery of phagocytic activity was also present in differentiated macrophages obtained from 5-FU/*E*O treated animals, compared to rats treated with 5-FU alone (Fig. 8e).

## Discussion

Some biological effects of *Eucalyptus *oil (*E*O) extract such as its antiseptic properties against a range of microbial agents [6,7] and, recently, its anti-inflammatory potential, both *in vitro *[15,24] and *in vivo *[16,17,25], mainly concerning the effect on some cytokine production, have been demonstrated. However, rather little is known about the influence of this essential oil on the cellular components of the immune system, and in particular on the monocytic/macrophagic system constituting one of the primary cellular effectors of the immune response against foreign particles and pathogen attacks.

In this study we present findings indicating that *E*O from *Eucalyptus globulus *is able to induce morphological and functional activation of human MDMs *in vitro*, dramatically stimulating the phagocytic response of these effectors of the innate immune defence. The acquisition of morphological features of *E*O activated macrophages and the increased phagocytic ability is coupled to a low release of IL-4, IL-6 and TNF-α pro-inflammatory cytokines, in contrast to that recorded under LPS stimulation.

The differences in LPS- or *E*O-induced cytokine production were associated with differences in the cytoskeletal elements that mediate phagocytosis, since *E*O-stimulated internalization requires integrity of the microtubule network, while LPS-stimulation does not. This suggests, almost preliminary, that LPS and *E*O might act by means of different mechanisms, possibly involving different phagocytic receptors. It is actually known that internalization by macrophages occurs by a restricted number of phagocytic receptors present on their surface [26]. Specifically, infectious agent are mainly phagocytosed by complement receptors (CR), after relatively non specific opsonization with complement, and by Fc receptors (FcR) after specific opsonization with antibodies. Important differences in the molecular mechanisms underlying phagocytosis by these two different receptors are actually recognized. These include differences in the cytoskeletal elements that mediate ingestion, differences in vacuole maturation, and differences in inflammatory responses. In particular, FcR-mediated phagocytosis is tightly coupled to the production and secretion of pro-inflammatory mediators such as cytokines and reactive oxygen intermediates [27], while CR-mediated phagocytosis is not. Furthermore, only the CR-mediated internalization requires integrity of the microtubular network [28]. On this context, our data indicate that *E*O might implement pathogens internalization stimulating the CR-mediated phagocytosis. *E*O-induced implementation of innate cell-mediated immune response, coupled to reduced production of pro-inflammatory cytokines and toxic oxygen intermediates, might have profound implications for the inflammatory response during pathogen infections.

The presence in the *E*O treated cultures of numerous phagocyting polarised cells, exhibiting elongated lamellopodia and filopodia, indicates that stimulation of pseudopodial activity and cell motility might contribute to the augmented phagocytic capability. The *in vitro *stimulatory effect was achieved using concentrations of the essential oil ranging from 0.008% and 0.016% (v/v), doses that resulted absolutely non toxic and up to 100 fold lower than those used *in vitro *by other authors [24,29].

Implementation of innate cell-mediated immune response has been observed not only in *in vitro *experiments but also in treated BDIX rats after 15 days *E*O administration. The stimulatory effect in treated animals, mainly involving the peripheral blood monocytes/granulocytes, led to a raise in the percentage of circulating monocytes as well as to an increment in the phagocytic activity of granulocytes and mostly of monocytic cells. These latter cells also exhibited an enhanced potential of extravasation ability, as suggested by increased expression of CD44 on their cellular membrane [18], as well as characteristics of activated monocytes [19,20], as indicated by the higher percentage of CD25^+ ^cells in circulating monocytes, compared to the untreated control rats.

The stimulatory effect on phagocytic ability recorded in circulating monocytes was also retained in monocyte-derived macrophages from treated animals, as showed by the *ex vivo *experiments. The *E*O dose used in the *in vivo *experiments (12 mg/Kg/die) did not produce evident toxic effect on treated rats, as suggested by the lack of body weight reduction and of animals mortality. This dose was comparable to doses recommended for oral therapy in humans and much more lower than dose that have been described to exhibit sub-acute toxicity in rats (about 300 mg/kg/die) [30].

Besides the effect of *E*O on the cell-mediated immune response in immuno-competent rats, this essential oil is also able to induce a dramatic recovery of granulocytes/monocytes activity after bone marrow suppression produced by 5-FU administration. In fact, combined treatment 5-FU/*E*O not only inhibited myelotoxicity, as resulted by the restoration of granulocytes number to normal values, but also raised the phagocytic activity of granulocytes and monocytes as well as of the *ex vivo *analyzed monocyte-derived macrophages, significantly decreased in animals treated with the chemotherapic alone. Since myelosuppression continues to be a major dose-limiting side effect for most chemotherapeutic agent such as 5-FU [23], our results suggest that the combination of *E*O with 5-FU might be taken into account for the prevention of immunotoxicity and myelotoxicity caused by 5-FU administration, even if additional studies should be carried out to demonstrate that the antitumor activity of 5-FU is not influenced by this combined therapeutic strategy.

Moreover, further experiments are in progress to determine whether the immuno-stimulatory effect, observed both *in vitro *and *in vivo*, could be due to the major monoterpenoid component eucalyptol (1,8-cineole), constituting the 60–90% of *Eucalyptus *oil [15], or is related to its synergic cooperation with the other minor components present in the crude extract; preliminary results seem to be indicative of this later hypothesis.

## Conclusion

Most knowledge of the therapeutic use of plant essential oils is acquired through folklore, and only some activities of these natural extracts are actually supported by scientific studies. But plants are an important source for drug discovery, and investigations on biological actions of plant medicinal extracts, as well as the understanding of the mechanisms underlying these actions drive the search for novel drugs.

Overall, our data, demonstrating that *Eucalyptus *essential oil from *Eucalyptus globulus *is able to implement the innate cell-mediated immune response, provide scientific support for an additional use of this plant extract, besides those concerning its known antiseptic and anti-inflammatory properties. Thus, the present study stimulates further investigations also using single components of essential oil extracts from various species of *Eucalyptus *for development of a possible new class of immuno-regulatory agents useful as adjuvant in immuno-suppressive pathologies, in infectious disease as well as in tumour chemotherapy.

## Methods

### Human monocyte derived macrophage (MDMs) cultures

Peripheral blood mononuclear cells (PBMCs) were isolated from buffy coats of healthy donors by density gradient centrifugation using Lympholyte-H (Cederlane, Hornby, Ontario, Canada). The lymphocytic/monocytic fraction was then re-suspended in RPMI 1640 medium (Hyclone Labs Inc. Logan, UTAH) supplemented with 10% (v/v) heat-inactivated fetal calf serum (FCS) (Hyclone), L-glutamine (2 mM), penicillin (100 IU/ml) and streptomycin (100 mg/ml) and cells were seeded on 175 cm^2 ^flasks and maintained at 37°C in 5% CO_2_, to generate adhering MDMs. After 1 h of culture, non-adhering cells were removed and the residual adhering MDMs were maintained in culture for 7 days to obtain partially differentiated macrophages. At this time, cells were detached using cold PBS, seeded at a density of 2.5 × 10^4 ^cells/cm^2 ^in 35 mm culture plates or on cover-slips (∅ 10 mm) and allowed to adhere for 4–5 days before treatments.

### MDMs *in vitro *treatments

Essential oil from *Eucalyptus globulus *was purchased from Cruciani Prodotti Crual^® ^Srl, (Rome, Italy). To exclude that the oil preparation used contained any endotoxins, we tested the *E*O extract using the Limulus Amebocyte Lysate (LAL) test (PYROGENT. Plus – Lonza Walkersville, Inc., Walkersville MD) The LAL test is a qualitative test for Gram-negative bacterial endotoxin utilizing a lysate prepared from the circulating amebocytes of the horseshoe crab (*Limulus polyphemus*) standardized to detect the labelled concentration (EU/ml) of the FDA Reference Standard Endotoxin. Limulus Amebocyte Lysate was mixed in equal parts with the solution being tested and incubated 1 h at 37°C. In the presence of endotoxin, a positive reaction is characterized by the formation of a firm gel; in the absence of endotoxin, gelation does not occur. The assay was done as a yes/no test. The EO samples and controls were diluted in sterile water, performed in triplicate and run in parallel. Positive controls consisted in 2 ng/ml (1 EU/ml) *E. coli *Control Standard Endotoxin or 0.1 μg/ml LPS; negative control consisted in sterile water. The LAL test excluded that the *E*O extract contained any endotoxins. (see Table S1 in the additional file 4).

Human MDMs were treated with 0.008% v/v in RPMI 1640 medium (corresponding to about 50 μg/ml) or 0.016% v/v (about 100 μg/ml) of *Eucalyptus *oil (*E*O) for 24 h. Concentrations were selected on the basis of the lowest, non toxic, effective doses found in a preliminary dose-response experiment (see Additional file 1). Cell viability after *E*O treatment was determined by the Trypan blue dye exclusion method. MDMs stimulated with 0.1 μg/ml of bacterial lipopolysaccharide (LPS, Sigma-Aldrich Co., St. Louis, Mo, USA) for 6 h were used as positive control of macrophage activation. The effect of *E*O 24 h pre-treatment before stimulation with LPS was also analysed. Essential oils from *Lavender *oil and the *Tea Tree *oil (Cruciani Prodotti Crual^® ^Srl), were used as controls to exclude a non specific effect on macrophages phagocytic activity caused by the oil preparation. For any microscopic analysis cells were grown on cover-slips.

### Optical and Scanning Electron Microscopy (SEM)

Analysis of morphological feature of activated MDMs, was carried out by phase-contrast microscopy after Wright Giemsa staining and by scanning electron microscopy (SEM). For SEM observation, MDMs were fixed with 2.5% glutharaldehyde in 0.1 M Millonig's phosphate buffer (MPB) at 4°C for 1 h. After washing in MPB, cells were post-fixed with 1% OsO_4 _in the same buffer for 1 h at 4°C and dehydrated using increasing acetone concentrations. The specimens were critical-point dried using liquid CO_2 _and sputter-coated with gold before examination on a Stereoscan 240 scanning electron microscope (Cambridge Instr., Cambridge, UK).

### Evaluation of phagocytic activity of human MDMs by confocal microscopy

The phagocytic activity of treated and untreated MDMs was tested by adding to cultures 2 × 10^7 ^beads/ml of yellow-green fluorescent polystyrene beads (∅ 1 μm, at a ratio of at least 10 beads/cell) with excitation/emission wavelengths of approximately 495 nm/515 nm (Molecular Probes, Inc., Eugene, OR). After 30 min, cells were fixed with paraformaldehyde, counter-stained with 1 μg/ml propidium iodide (PI – Sigma-Aldrich) and observed by the confocal microscope LEICA TCS SP5 (Leica Instruments, Heidelberg, Germany). The excitation/emission wavelengths employed for PI staining were 568 nm/590 nm. A minimum of 500 cells per sample were observed, the number of phagocytic MDMs (reported as percent of phagocytic cells), as well as the number of beads per cell, were counted. MDMs subjected to beads administration and maintained at 0°C to block internalization, was used as negative control of uptake. Quantitative assessment was done in a blinded fashion. The experiment was repeated three times and the mean values were plotted. We analyzed at least 20 buffy coats from different healthy donors.

### Evaluation of cytokines production

The concentration of IL-2, IL-4, IL-6, IL-10, TNF-α, and IFN-γ secreted into the culture media by human macrophages after *in vitro E*O treatment were determined by using the BD Cytometric Bead Array human Th1/Th2 cytokine kit (BD Pharmingen) according to the manufacturer's protocol [31]. Flow cytometry analysis was carried out using a FACSCalibur flow cytometer (Becton Dickinson, Mountain View, CA). The effect of *E*O pre-treatment on the pro-inflammatory and immune modulating cytokines production by macrophages stimulated with LPS was also evaluated.

### *In vitro *inhibitory study

To depolymerize microtubules, control cells and macrophages, pre-treated with LPS or *E*O, were treated with 2 μg/ml nocodazole for 30 min. For phagocytic activity testing, after nocodazole treatment, fluorescent polystyrene beads were added to the cell culture, samples were processed as described above and analysed by confocal microscopy. The effect of nocodazole on microtubular network was analysed after immuno-staining, using an anti-human tubulin mouse monoclonal antibody (Molecular Probes) revealed with the secondary Alexa Fluor 488-conjugated anti-mouse IgG (Molecular Probes), by confocal microscopy. Cells were counter-stained with PI (Sigma-Aldrich).

### Animals and *in vivo *treatments

Inbred male BDIX rats (Charles River, Calco, Italy), 8 weeks old and weighing 220–250 g, were held for 7 days, housed in a pathogen-free animal facility with free access to water and standard food and kept in accordance with European Community guidelines. Experiments were approved by the local committee on animal experimentation, and were performed under strict governmental and international guidelines. *E*O was administrated for 15 days *per os*, at a dose of 12 mg/Kg/day, by adding, every evening, the essential oil extract directly to drinking water (7 μl/day of essential oil per animal). Two sets of *in vivo *experiments were carried out. In the first one, in which we tested the effect of *E*O on peripheral blood mononuclear cells of immuno-competent rats, animals were divided in two groups (12 animals/group), an untreated control group and a group treated for 15 days with *E*O (see schematic diagram in Fig. 6a). In a second experiment, in which we tested whether *E*O treatment could be able to induce a recovery of peripheral blood mononuclear cells activity after bone marrow suppression, rats of both groups (12 animals/group) were intra-peritonally injected with a single dose of 100 mg/Kg of the chemotherapeutic agent 5-fluorouracil (5-FU) on day 7 from the beginning of *E*O treatment (see schematic diagram in Fig. 8a). In both sets of experiments, from all animals, anaesthetized by inhalation of 2-bromo-2-chloro-1,1,1-trifluoroethane (Fluka, Sigma-Aldrich), blood was collected, by intracardiac puncture, before treatment (day 0 – T_0_), on day 7 (T_7_), on day 15 (T_15_) and on day 20 (T_20_). For evaluation of haematological parameters, after erythrocytes lysis by FACS Lysing Solution (Becton Dickinson), mononuclear cell fractions in RPMI 1640 medium were analyzed for forward (FSC) and sideward (SSC) scatter patterns in a fluorescence-activated cell sorter (FACScan, Becton Dickinson). Gates were defined to identify populations of cells with different FSC/SSC characteristics, corresponding to granulocytes, lymphocytes or monocytes populations and results were expressed as percent of total cells. In addition, rat peripheral blood mononuclear cells (PBMCs) were isolated by density gradient centrifugation using Lympholyte-H (Cederlane, Hornby, Ontario, Canada). The lymphocytic/monocytic fraction was then re-suspended in RPMI 1640 medium and rat adhering macrophages were obtained as described previously for human MDMs and used for the *ex vivo *experiments.

### Evaluation of phagocytic activity of rat peripheral mononuclear cells and macrophages

The evaluation of phagocytic activity of monocytes/granulocytes from peripheral blood of BDIX rats, after *in vivo EO *administration, in absence or in presence of bone marrow suppression by 5-FU administration, was carried out by cytofluorimetric analysis using the phagotest kit (ORPEGEN Pharma, Heidelberg, Germany), following the manual's protocol. This test allows the quantitative determination of leukocyte phagocytosis in heparinized whole blood. It contains fluorescein (FITC)-labelled opsonized bacteria (*E. coli*-FITC) and necessary reagents, and measures the overall percentage of phagocytic monocytes and granulocytes (that have ingested one or more bacteria per cell) and the individual cellular phagocytic activity (number of bacteria per cell) recorded as Mean Fluorescence Intensity (MFI).

The phagocytic activity of rat MDMs, obtained from treated and untreated animals and grown on cover-slips, was evaluated *ex vivo *by confocal microscopy after fluorescent beads administration, as previously described for the *in vitro *experiments on human MDMs. To test the phagocytic ability towards microbial pathogens, MDMs from treated and untreated immuno-competent animals were subjected to an *in vitro *infection with a suspension of *Staphylococcus aureus *(1 × 10^5^bacteria/cell), and observed after 6 h of culture by confocal microscopy.

### Evaluation of CD44 expression and CD25^+ ^cells in circulating monocytes

The expression of CD44 and the percentage of CD25^+ ^cells in circulating monocytes from treated and untreated rats were evaluated by cytofluorimetric analysis. Living cells were incubated with a mouse anti-rat CD44H (clone OX-49; BD Pharmingen), detected using a PE-conjugated polyclonal anti-mouse IgG (BD Pharmingen) or with a FITC-conjugated mouse anti-rat CD25 (clone OX-39; BD Pharmingen), using a FACScan flow cytometer (Becton Dickinson).

### Statistical analysis

For statistical analysis the two-tailed Student's *t *test was used. For the *in vitro *evaluations, at least three independent experiments have been carried out and data are given as the mean ± SD. For the *in vivo *and *ex vivo *experiments, results are reported as mean of 12 animals/group ± SD. For all analyses, significance was calculated with a *P *value < 0.01.

## Abbreviations

*E*O: *Eucalyptus *oil; 5-FU: 5-fluorouracil; *Lav*O: *Lavender *oil; LPS: bacterial lipopolysaccharide; MDMs: monocyte-derived macrophages; MPB: Millonig's phosphate buffer; PBMCs: peripheral blood mononuclear cells; SEM: scanning electron microscopy; *TeaTree*O: *Tea Tree *oil.

## Authors' contributions

AS conceived of the study and designed the experiments, carried out all microscopic analyses, collected and interpreted data, drafted the manuscript. PSV has been involved in drafting the manuscript and revising it critically. FA performed the macrophage cultures and treatment, carried out preparation of samples for confocal and electron microscopy observations, participated in the *in vivo *study. MZ carried out the *in vivo *study, and the preparation of samples for cytofluorimetric analysis, participated in the macrophage cultures maintenance and treatment. LM participated in the *in vivo *study, and in the macrophage cultures maintenance and treatment. MF participated in the design of the study and has been involved in revising the manuscript critically. GR has been involved in drafting the manuscript and revising it critically. EG has been involved in revising the manuscript critically. PP participated in the design of the experiments, carried out all cytofluorimetric analysis, and has been involved in drafting the manuscript and revising it critically.

## Supplementary Material

Additional file 1 Dose-response experiment performed to select the lowest, non toxic, effective doses of *Eucalyptus *oil used in the *in vitro *studies. The data provided report the cell survival and the phagocytic activity of MDMs after 24 h treatment with increasing concentrations of *E*O Click here for file

Additional file 2 *In vitro *effect of *Lavender *oil (*Lav*O) or *Tea Tree *oil (*TeaTree*O) treatments on phagocytic activity of human MDMs. The data provided show the phagocytic activity of MDMs treated for 24 h with 0.008% and 0.016% *Lav*O or *TeaTree*O, compared to *E*O used at the same concentrations. Click here for file

Additional file 3 *In vitro *effect of *Lav*O or *TeaTree*O treatments on viability of human MDMs. The data provided show the viability of MDMs treated for 24 h with 0.008% and 0.016% *Lav*O or *TeaTree*O, compared to *E*O used at the same concentrations. Click here for file

Additional file 4 Endotoxin detection by Limulus Amebocyte Lysate (LAL) test. The data, provided in a Table, show the results of LAL test performed on EO extract used in this study and in negative (sterile water) or positive (E. coli Control Standard Endotoxin or LPS) controls. Click here for file

## References

[B1] Stafford JL, Neumann NF, Belosevic M (2002). Macrophage-mediated innate host defense against protozoan parasites. Crit Rev Microbiol.

[B2] Underhill DM, Ozinsky A (2002). Phagocytosis of microbes: complexity in action. Annu Rev Immunol.

[B3] Gougerot-Pocidalo MA, el Benna J, Elbim C, Chollet-Martin S, Dang MC (2002). Regulation of human neutrophil oxidative burst by pro- and anti-inflammatory cytokines. Soc Biol.

[B4] Harkenthal M, Layh-Schmitt G, Reichling J (2000). Effect of Australian tea tree oil on the viability of the wall-less bacterium Mycoplasma pneumoniae. Pharmazie.

[B5] Cox SD, Mann CM, Markham JL (2001). Interactions between components of the essential oil of Melaleuca alternifolia. J Appl Microbiol.

[B6] Schnitzler P, Schon K, Reichling J (2001). Antiviral activity of Australian tea tree oil and eucalyptus oil against herpes simplex virus in cell culture. Pharmazie.

[B7] Takarada K, Kimizuka R, Takahashi N, Honma K, Okuda K, Kato T (2004). A comparison of the antibacterial efficacies of essential oils against oral pathogens. Oral Microbiol Immunol.

[B8] Messager S, Hammer KA, Carson CF, Riley TV (2005). Assessment of the antibacterial activity of tea tree oil using the European EN 1276 and EN 12054 standard suspension tests. J Hosp Infect.

[B9] Brand C, Grimbaldeston MA, Gamble JR, Finlay-Jones JJ, Hart PH (2002). Tea tree oil reduces the swelling associated with the efferent phase of a contact hypersensitivity response. Inflamm Res.

[B10] Hart PH, Brand C, Carson CF, Riley Tv, Prager RH, Finlay-Jones JJ (2000). Terpinen-4-ol, the main component of the essential oil of Melaleuca alternifolia (tea tree oil), suppresses inflammatory mediator production by activated human monocytes. Inflamm Res.

[B11] Kim H-M, Cho S-H (1999). Lavender oil inhibits immediate-type allergic reaction in mice and rats. J Pharm Pharmacol.

[B12] Brand C, Townley SL, Finlay-Jones JJ, Hart PH (2002). Tea tree oil reduces histamine-induced oedema in murine ears. Inflamm Res.

[B13] Santos FA, Rao VSN (1997). Mast cell involvement in the rat paw oedema response to 1,8-cineole, the main constituent of eucalyptus and rosemary oils. Eur J Pharmacol.

[B14] Brand C, Ferrante A, Prager RH, Riley TV, Carson CF, Finlay-Jones JJ, Hart PH (2001). The water-soluble components of the essential oil of Melaleuca alternifolia (tea tree oil) suppress the production of superoxide by human monocytes, but not neutrophils, activated in vitro. Inflamm Res.

[B15] Juergens UR, Stober M, Vetter H (1998). Inhibition of cytokine production and arachidonic acid metabolism by eucalyptol (1,8-cineole) in human blood monocytes in vitro. Eur J Med Res.

[B16] Juergens UR, Stober M, Schmidt-Schilling L, Kleuver T, Vetter H (1998). Antiinflammatory effects of eucalyptol (1,8-cineole) in bronchial asthma: inhibition of arachidonic acid metabolism in human blood monocytes ex vivo. Eur J Med Res.

[B17] Juergens UR, Dethlefsen U, Steinkamp G, Gillissen A, Repges R, Vetter H (2003). Anti-inflammatory activity of 1.8-cineol (eucalyptol) in bronchial asthma: a double-blind placebo-controlled trial. Respir Med.

[B18] Siegelman MH, DeGrendele HC, Estess P (1999). Activation and interaction of CD44 and hyaluronan in immunological systems. J Leukocyte Biol.

[B19] Espinoza-Delgado I, Longo DL, Gusella GL, Varesio L (1992). Regulation of IL-2 receptor subunit genes in human monocytes. Differential effects of IL-2 and IFN-gamma. J Immunol.

[B20] Sanarico N, Ciaramella A, Sacchi A, Bernasconi D, Bossù P, Mariani F, Colizzi V, Vendetti S (2006). Human monocyte-derived dendritic cells differentiated in the presence of IL-2 produce proinflammatory cytokines and prime Th1 immune response. J Leukocyte Biol.

[B21] Heidelberg C, Ansfield FJ (1963). Experimental and clinical use of fluorinated pyrimidines in cancer chemotherapy. Cancer Res.

[B22] Seifert P, Baker HL, Reed ML (1975). Comparison of continuously infused 5-fluorouracil with bolus injection in treatment of patients with colorectal adenocarcinoma. Cancer.

[B23] Schetz JD, Wallance HJ, Diasio RB (1984). 5-Fluorouracil incorporation into DNA of CF-1 mouse bone marrow cells as a possible mechanism of toxicity. Cancer Res.

[B24] Vigo E, Cepeda A, Gualillo O, Perez-Fernandez R (2004). In-vitro anti-inflammatory effect of *Eucalyptus globulus *and *Thymus vulgaris*: nitric oxide inhibition in J774A.1 murine macrophages. Pharm Pharmacol.

[B25] Silva J, Abebe W, Sousa SM, Duarte VG, Machado MIL, Matos FJA (2003). Analgesic and anti-inflammatory effects of essential oils of Eucalyptus. J Ethnopharmacol.

[B26] Aderem A, Underhill DM (1999). Mechanisms of phagocytosis in macrophages. Annu Rev Immunol.

[B27] Wright SD, Silverstein SC (1983). Receptors for C3b and C3bi promote phagocytosis but not the release of toxic oxygen from human phagocytes. J Exp Med.

[B28] Allen LAH, Aderem A (1996). Molecular definition of distinct cytoskeletal structures involved in complement- and Fc receptor-mediated phagocytosis in macrophages. J Exp Med.

[B29] Abe S, Maruyama N, Hayama K, Ishibashi H, Inoue S, Oshima H, Yamaguchi H (2003). Suppression of tumor necrosis factor-alpha-induced neutrophil adherence responses by essential oils. Mediators Inflamm.

[B30] De Vincenti M, Silano M, De Vincenzi A, Maialetti F, Scazzocchio B (2002). Constituents of aromatic plants: eucalyptol. Fitoterapia.

[B31] Rodriguez-Caballero A, Garcia-Montero AC, Bueno C, Almeida J, Varro R, Chen R, Pandiella A, Orfao A (2004). A new simple whole blood flow cytometry-based method for simultaneous identification of activated cells and quantitative evaluation of cytokines released during activation. Lab Invest.

