# Supplementary material for: Stimulatory effect of Eucalyptus essential oil on innate cell-mediated immune response
Source: BMC Immunol. 2008 Apr 18;9:17. doi: 10.1186/1471-2172-9-17 (PMC2374764; doi:10.1186/1471-2172-9-17)
Supplement: Additional file 1 — Dose-response experiment performed to select the lowest, non toxic, effective doses of Eucalyptus oil used in the in vitro studies. The data provided report the cell survival and the phagocytic activity of MDMs after 24 h treatment with increasing concentrations of EO [file 1471-2172-9-17-S1.pdf]

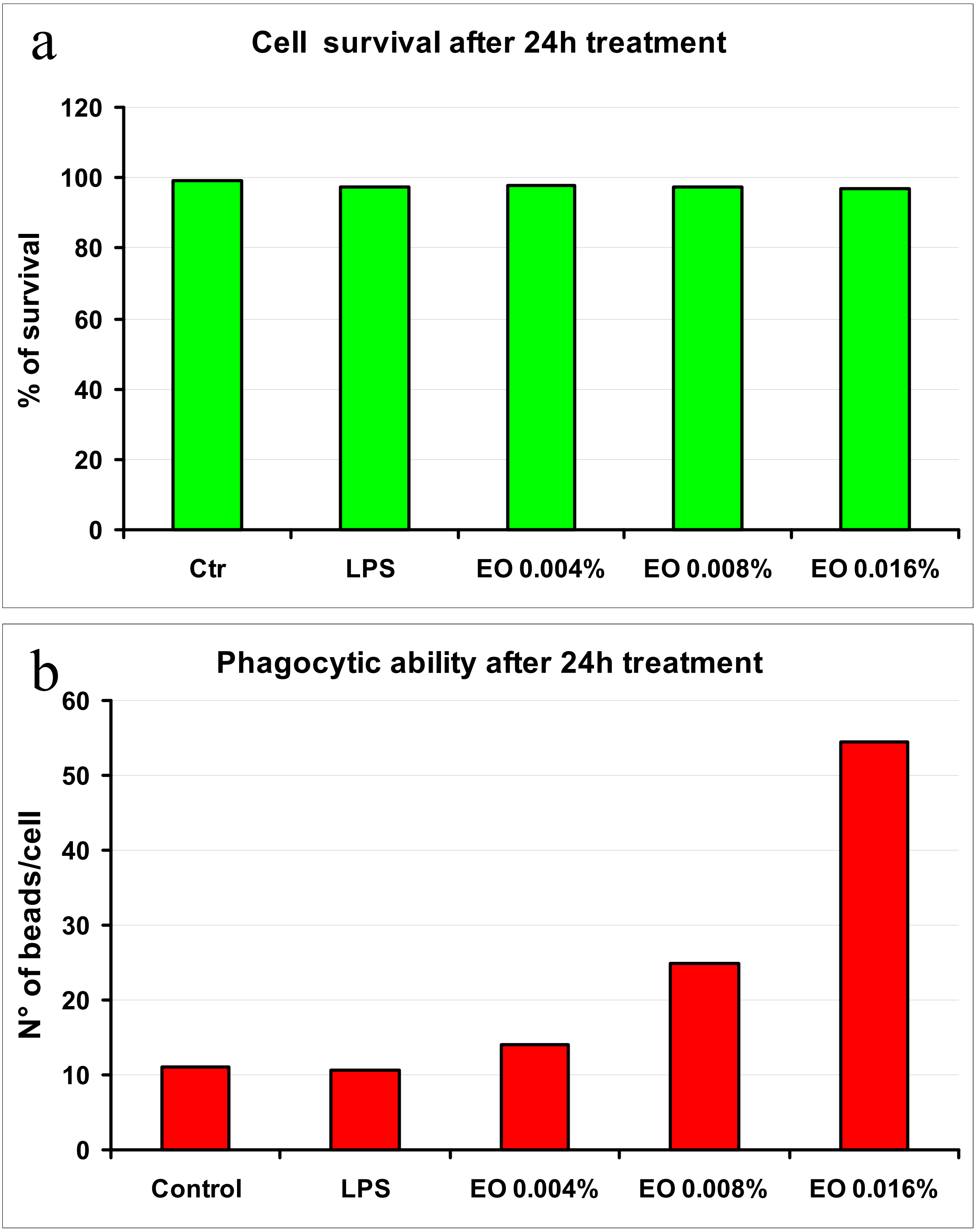
a
Cell survival after 24h treatment
% of survival
120
100
80
60
40
20
0
Ctr
LPS
EO 0.004%
EO 0.008%
EO 0.016%
b
Phagocytic ability after 24h treatment
N° of beads/cell
60
50
40
30
20
10
0
Control
LPS
EO 0.004%
EO 0.008%
EO 0.016%

Figure S1

Figure legend

Figure S1.

Dose-response experiment performed to select the lowest, non toxic, effective doses of *Eucalyptus* oil used in the *in vitro* studies. a, MDMs viability after 24h treatment with increasing concentrations of *E*O determined by the Trypan blue dye exclusion method. Cell survival of MDMs culture stimulated with 0.1μg/ml of LPS is also reported. The results, reported as percentage of survival, are the mean of three different experiments. **b,** phagocytic activity of MDMs treated with increasing concentration of *E*O evaluated by adding to cultures $2x10^7$ beads/ml of fluorescent polystyrene beads. MDMs phagocytic activity of culture stimulated with LPS is also reported. A minimum of 500 cells per sample were observed and results are reported as number of beads per cell.
