# Supplementary material for: Stimulatory effect of Eucalyptus essential oil on innate cell-mediated immune response
Source: BMC Immunol. 2008 Apr 18;9:17. doi: 10.1186/1471-2172-9-17 (PMC2374764; doi:10.1186/1471-2172-9-17)
Supplement: Additional file 2 — In vitro effect of Lavender oil (LavO) or Tea Tree oil (TeaTreeO) treatments on phagocytic activity of human MDMs. The data provided show the phagocytic activity of MDMs treated for 24 h with 0.008% and 0.016% LavO or TeaTreeO, compared to EO used at the same concentrations. [file 1471-2172-9-17-S2.pdf]

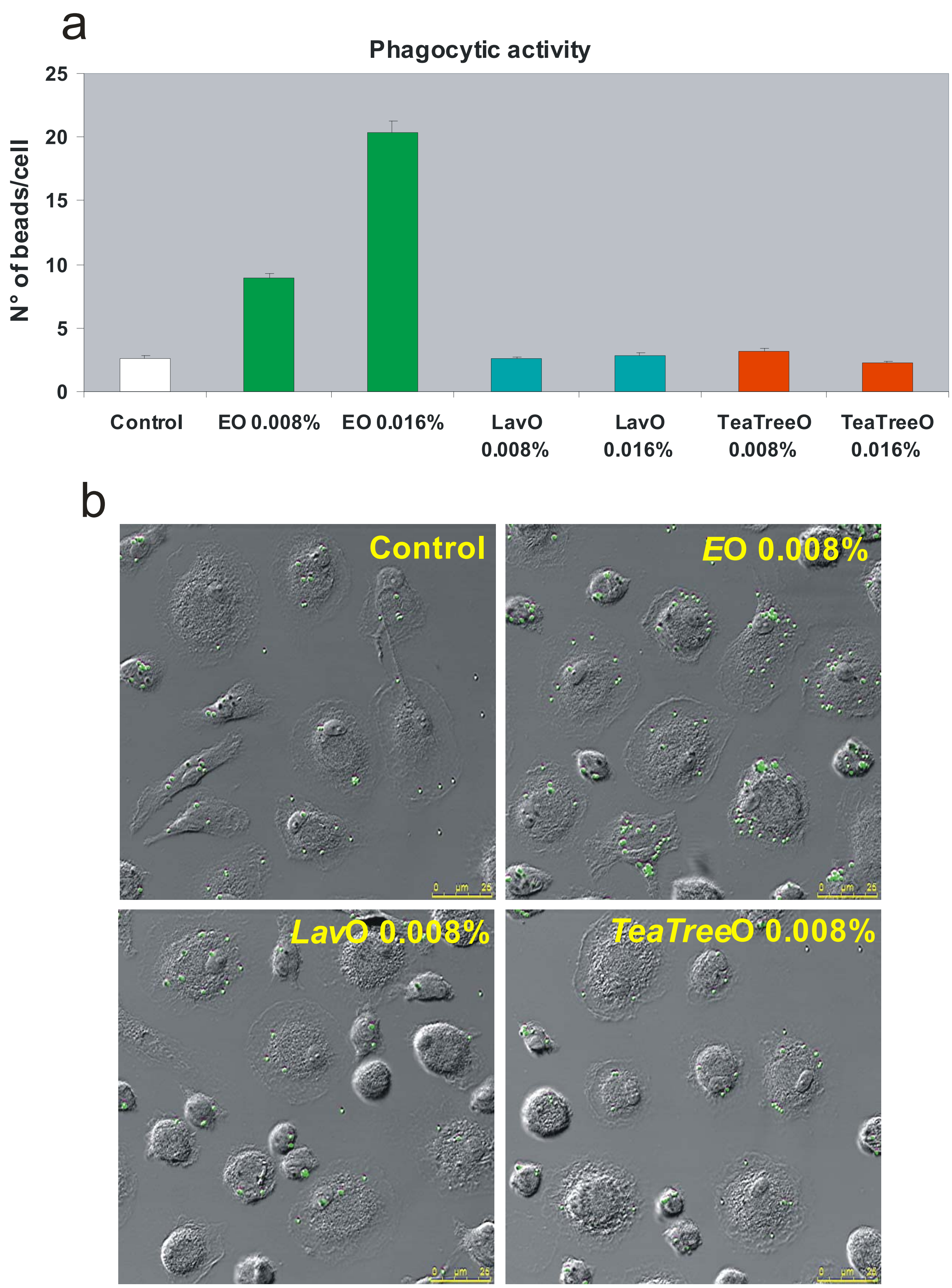
a
Phagocytic activity
N° of beads/cell
25
20
15
10
5
0
Control
EO 0.008%
EO 0.016%
LavO 0.008%
LavO 0.016%
TeaTreeO 0.008%
TeaTreeO 0.016%
b
Control
EO 0.008%
LavO 0.008%
TeaTreeO 0.008%

Figure S2

Figure legend

Figure S2.

***In vitro* effect of *Lavender* oil (*Lav*O) or *Tea Tree* oil (*TeaTree*O) treatments on phagocytic activity of human MDMs compared to *Eucalyptus* oil. a**, bar graph showing the phagocytic activity of MDMs treated for 24h with 0.008% and 0.016% *Lav*O or *TeaTree*O, compared to *E*O used at the same concentrations. Results are reported as number of beads per cell. **b**, representative images, by confocal microscopy, of untreated control cells and MDMs cultures treated for 24h with 0.008% *E*O, *Lav*O or *TeaTree*O: cell morphology was visualized by differential interference contrast while fluorescent signal of beads was visualized by green hue. Note that the number of beads/cell in *E*O treated cultures is higher than the control, while in *Lav*O or *TeaTree*O treated cells it is similar to the untreated control.
