# Supplementary material for: Stimulatory effect of Eucalyptus essential oil on innate cell-mediated immune response
Source: BMC Immunol. 2008 Apr 18;9:17. doi: 10.1186/1471-2172-9-17 (PMC2374764; doi:10.1186/1471-2172-9-17)
Supplement: Additional file 3 — In vitro effect of LavO or TeaTreeO treatments on viability of human MDMs. The data provided show the viability of MDMs treated for 24 h with 0.008% and 0.016% LavO or TeaTreeO, compared to EO used at the same concentrations. [file 1471-2172-9-17-S3.pdf]

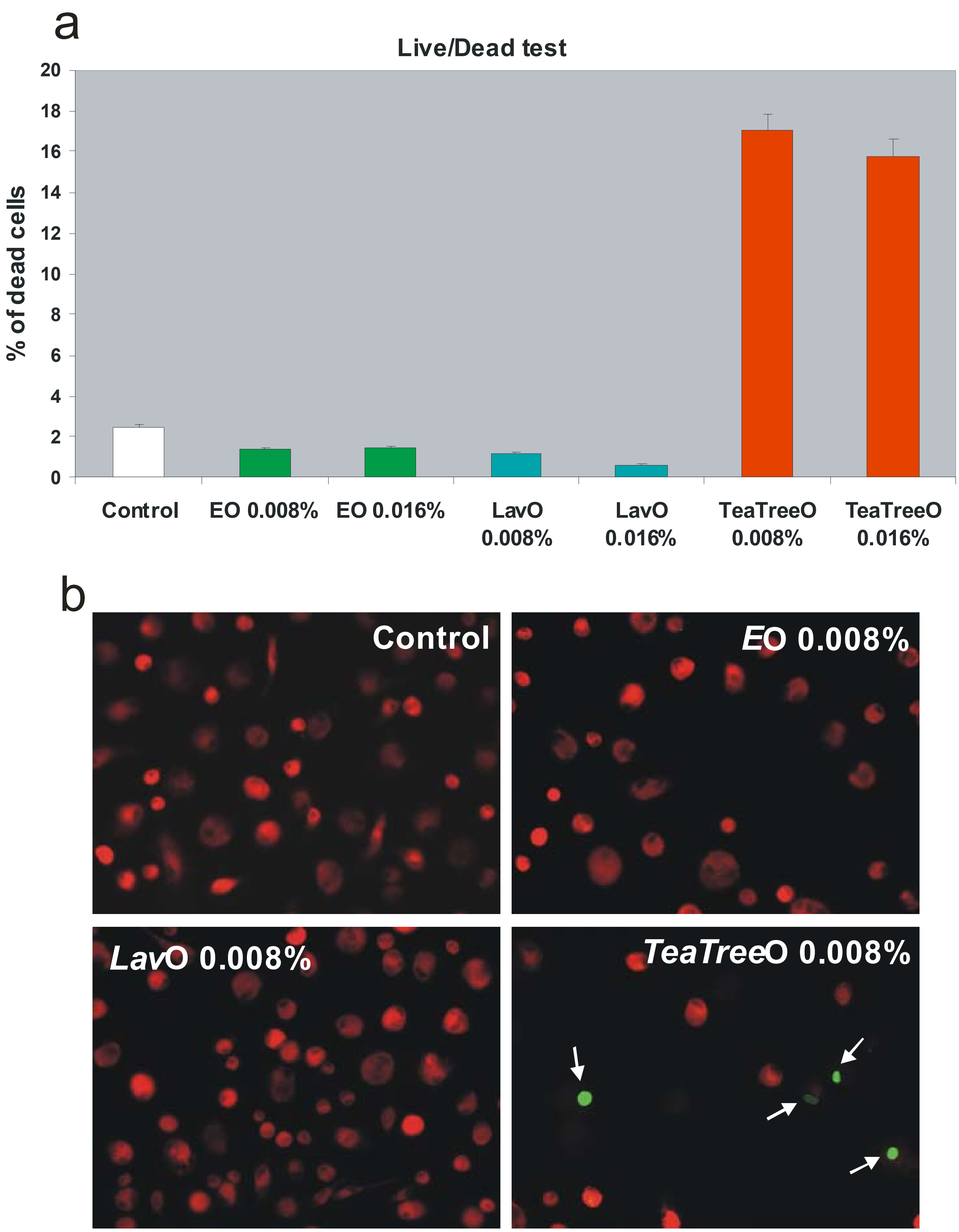
a
Live/Dead test
% of dead cells
20
18
16
14
12
10
8
6
4
2
0
Control
EO 0.008%
EO 0.016%
LavO 0.008%
LavO 0.016%
TeaTreeO 0.008%
TeaTreeO 0.016%
b
Control
EO 0.008%
LavO 0.008%
TeaTreeO 0.008%

Figure S3

Figure legend

Figure S3.

***In vitro* effect of *Lavender* oil (*Lav*O) or *Tea Tree* oil (*TeaTree*O) treatments on viability of human MDMs compared to *Eucalyptus* oil.** Cell toxicity was evaluated by confocal microscopy (CLSM) using the two-color fluorescence Live/Dead Viability/Cytotoxicity assay (Molecular Probes, Inc., Eugene, OR) that distinguishes metabolically active cells (cytoplasmic red hue) from injured cells and dead cells (nuclear green hue). **a**, Bar graph showing viability of MDMs treated for 24h with 0.008% and 0.016% *Lav*O or *TeaTree*O, compared to *E*O used at the same concentrations. A minimum of 300 cells per sample were observed, the number of live or dead cells were counted and reported as percent of dead cells. Quantitative assessment was done in a blinded fashion. **b**, representative images, by confocal microscopy, of untreated control cells and MDMs cultures treated for 24h with 0.008% *E*O, *Lav*O or *TeaTree*O: arrows point to dead cells in *TeaTree*O treated cultures.
