# Supplementary material for: Stimulatory effect of Eucalyptus essential oil on innate cell-mediated immune response
Source: BMC Immunol. 2008 Apr 18;9:17. doi: 10.1186/1471-2172-9-17 (PMC2374764; doi:10.1186/1471-2172-9-17)
Supplement: Additional file 4 — Endotoxin detection by Limulus Amebocyte Lysate (LAL) test. The data, provided in a Table, show the results of LAL test performed on EO extract used in this study and in negative (sterile water) or positive (E. coli Control Standard Endotoxin or LPS) controls. [file 1471-2172-9-17-S4.pdf]

Table S1. Endotoxin detection by Limulus Amebocyte Lysate (LAL) test

	**1**	**2**	**3**	**Results**
Sterile water	–	–	–	Negative
LPS 0.1µg/ml	+	+	+	Positive
E. Coli CSE 2ng/ml (1EU/ml)	+	+	+	Positive
Eucalyptus Oil 0.008%	–	–	–	Negative
Eucalyptus Oil 0.016%	–	–	–	Negative

The *Eucalyptus* oil extract was tested using the Limulus Amebocyte Lysate (LAL) test. The assay was done as a yes/no test. The *Eucalyptus* oil samples and controls were diluted in sterile water, performed in triplicate and run in parallel. Positive controls consisted in 2ng/ml (1EU/ml) *E. coli* Control Standard Endotoxin (CSE) or 0.1µg/ml LPS; negative control consisted in sterile water. In EO extract, tested at both 0.008% and 0.016% concentrations used for the *in vitro* treatment, we did not recorded any endotoxin content.
